# Extensive Study of Breast Milk and Infant Growth: Protocol of the Cambridge Baby Growth and Breastfeeding Study (CBGS-BF)

**DOI:** 10.3390/nu13082879

**Published:** 2021-08-21

**Authors:** Laurentya Olga, Clive J. Petry, Janna A. van Diepen, Philippa M. Prentice, Ieuan A. Hughes, Jacques Vervoort, Jos Boekhorst, Maciej Chichlowski, Gabriele Gross, David B. Dunger, Ken K. Ong

**Affiliations:** 1 Department of Paediatrics, University of Cambridge, Cambridge CB2 0QQ, UK; lo290@medschl.cam.ac.uk (L.O.); cjp1002@medschl.cam.ac.uk (C.J.P.); philippaprentice@doctors.org.uk (P.M.P.); iah1000@medschl.cam.ac.uk (I.A.H.); dbd25@cam.ac.uk (D.B.D.); 2 Department of Internal Medicine and Radboud Institute for Molecular Life Sciences, Radboud University Medical Center, 6545 CJ Nijmegen, The Netherlands; Janna.vanDiepen@rb.com (J.A.v.D.); Gabriele.Gross@rb.com (G.G.); 3 Laboratory of Biochemistry, Wageningen University, 6708 PB Wageningen, The Netherlands; jacques.vervoort@wur.nl; 4 NIZO Food Research BV, 6718 ZB Ede, The Netherlands; Jos.Boekhorst@nizo.com; 5 Department of Medical Affairs, Mead Johnson Nutrition, Evansville, IN 47721, USA; Maciej.Chichlowski@rb.com; 6 Wellcome-MRC Institute of Metabolic Science, University of Cambridge, Cambridge CB2 0QQ, UK; 7 MRC Epidemiology Unit, University of Cambridge, Cambridge CB2 0SL, UK

**Keywords:** infant growth, breast milk, early life, cohort profile, infant nutrition, breast milk nutrients, human milk oligosaccharides, breastfeeding, childhood obesity, prevention

## Abstract

Growth and nutrition during early life have been strongly linked to future health and metabolic risks. The Cambridge Baby Growth Study (CBGS), a longitudinal birth cohort of 2229 mother–infant pairs, was set up in 2001 to investigate early life determinant factors of infant growth and body composition in the UK setting. To carry out extensive profiling of breastmilk intakes and composition in relation to infancy growth, the Cambridge Baby Growth and Breastfeeding Study (CBGS-BF) was established upon the original CBGS. The strict inclusion criteria were applied, focusing on a normal birth weight vaginally delivered infant cohort born of healthy and non-obese mothers. Crucially, only infants who were exclusively breastfed for the first 6 weeks of life were retained in the analysed study sample. At each visit from birth, 2 weeks, 6 weeks, and then at 3, 6, 12, 24, and 36 months, longitudinal anthropometric measurements and blood spot collections were conducted. Infant body composition was assessed using air displacement plethysmography (ADP) at 6 weeks and 3 months of age. Breast milk was collected for macronutrients and human milk oligosaccharides (HMO) measurements. Breast milk intake volume was also estimated, as well as sterile breastmilk and infant stool collection for microbiome study.

## 1. Introduction

The original Cambridge Baby Growth Study (CBGS) was set up in 2001, historically to investigate the effect of environmental factors on male reproductive development but then the entire birth cohort (males and females) were included to examine the ante- and postnatal determinants of infant growth and body composition, including genetic and environmental factors [1]. The recruitment took place until 2009 among pregnant mothers from a single maternity hospital in Cambridge. The study visits were conducted twice during pregnancy and four times postnatally at 3, 12, 18, and 24 months. The original CBGS has provided valuable insights into the maternal-foetal communication and pregnancy comorbidities [2,3], infant growth and nutrition [4,5,6,7] and its association to later childhood outcomes [8], as well as growth and adiposity development of infants at risk [9].

The original CBGS and other studies have associated breastfeeding with slower subsequent growth and adiposity gains in infancy and childhood compared to formula feeding [4,10,11,12], and thus support breastfeeding as a potential component in the early prevention against later obesity. It is conjectured that this difference in early growth rates may be due to the nutrient contents in human breast milk (BM). Accordingly, triglycerides, lactose, protein, and SCFA contents in BM have been considered important for subsequent infants’ weight and adiposity gains [4,5,13]. However, BM nutrient concentrations do not necessarily reflect the amount consumed by infants. The measurement of infant’s nutritional intake from BM could provide a better mechanistic link between breastfeeding and infancy growth and adiposity. Additionally several studies have reported that the establishment of infant gut microbiota, which is associated with the BM microbiome and potentially with BM oligosaccharide composition, may influence childhood weight gain trajectories, early metabolic programming, and hence later obesity risk [14,15].

Therefore, the CBGS-Breastfeeding Study (CBGS-BF) was established in 2015 in collaboration with the research division of Mead Johnson Nutrition, with the particular aim to identify factors in BM that are associated with infant growth and might reduce obesity risk later in life. In this study, parameters of BM intake and composition were studied more extensively, including BM intake volume using a deuterium-labelled water technique, longitudinal BM collection and a more detailed BM composition including macronutrients, butyrate and human milk oligosaccharides (HMOs), and explorative analyses of microbiota in BM and infant gut.

This study has been funded by Mead Johnson Nutrition with additional support from MRC Epidemiology Unit, NIHR Clinical Research Network, NIHR Cambridge Biomedical Research Centre, Newlife, and Mothercare.

## 2. Materials and Methods

Mother–infant pairs were recruited at birth from the same single centre as the original CBGS, the Rosie Maternity Hospital, Cambridge (UK). Inclusion criteria were: healthy term vaginally delivered singletons of normal birthweight (defined as greater than −1.5 SDS for gestational age, using British 1990 growth reference) and if the family intended to continue exclusive breastfeeding from birth until at least age 6 weeks. To allow for standardised microbiota sampling, further exclusion criteria were: maternal pre-pregnancy body mass index (BMI) > 30 kg/m^2^), any significant maternal illness or pregnancy comorbidity, use of antibiotics or steroids in 30 days before delivery, and regular consumption of probiotics. In total, 150 mother–infant pairs were recruited, of whom 94 were exclusively breastfed for at least 6 weeks and were thus eligible for retention in the study. During that exclusive breastfeeding period, as defined by the WHO, infants received solely BM and no other liquid or solid food was given, except drops of multivitamin/mineral supplements or medicines if indicated [16]. The CBGS-BF was approved by the National Research Ethics Service Cambridgeshire 2 Research Ethics Committee, and all mothers gave informed written consent.

**Figure 1:**
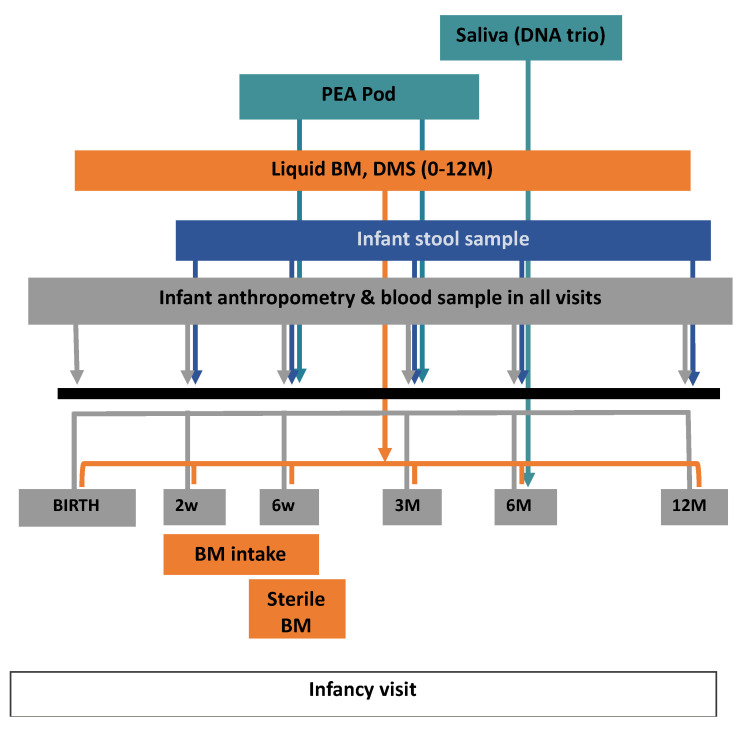
CBGS-BF study design. BM: breast milk, DMS: dried milk spot.

This study built on the same design and protocol as the original CBGS [1] with extra visits and biological sample collections, including infant stool, dried milk spot (DMS), and both maternal and infant urine for BM intake volume measurement (Table 1, Figure 1). There was also an addition to body composition measurement by estimating infant total body fat- and fat-free mass. This was conducted using air-displacement plethysmography (ADP), Pea Pod system (Life measurement Inc., Concord, CA, USA).

### 2.1. Growth and Adiposity Measurement

All anthropometry and body composition measurements were performed by three trained paediatric research nurses.

Birthweight was taken from routine medical records of measurements by health professionals at delivery. Other birth measurements (Table 1) were conducted by the research team in the first 8 days of life. At all subsequent visits, weight, length, subcutaneous skinfolds, head circumference, and waist circumference were measured by the research team (Figure 1, Table 1). All of these measurements were done in triplicate and averaged.

A Seca 757 electronic baby scale (Seca Ltd., Hamburg, Germany) was used to measure infant weight to the nearest 1 g. Infants were weighed before feeding, naked without diapers, or alternatively the weight of the diaper was subtracted from the measured weight. A Seca 416 infantometer was used to measure supine length to the nearest 0.1 cm. Weight and length measurements were used to calculate body mass index (BMI) and ponderal index (PI), a more accurate adiposity parameter during infancy, by dividing weight (kg) by length cubed (kg/m^3^). To measure head circumference (HC), a Seca 212 measuring tape was circled around the largest circumference of the head, i.e., from above the eyebrows and around the back of the head.

Adiposity measurements included subcutaneous skinfolds thicknesses, waist circumference (WC) and abdominal fat thickness (U/S), and accurate body composition estimation using air-displacement plethysmography (ADP)-Pea Pod.

Skinfold thicknesses were measured at four sites, triceps, subscapular, flank, and quadriceps, using a Holtain Tanner/Whitehouse Skinfold Caliper (Holtain Ltd., Crymych, Wales, UK). Triceps skinfold was measured at the posterior surface of the arm, halfway between the acromial process (shoulder) and the olecranon (elbow); subscapular skinfold at the oblique angle below the scapula (upper back); flank skinfold in the posterior axillary line immediately posterior to the iliac crest, and quadriceps skinfold in the midline and halfway between the top of the patella and the inguinal crease.

WC was measured using a Seca 201 ergonomic circumference measuring tape. WC was taken at the end of a normal expiration midway between the lowest rib and the iliac crest as the minimum diameter, preferably before feeding. A standard ultrasound machine (Logiq Book XP ultrasound with 3C MHZ-RS abdominal curved array transducer, GE Healthcare, Bedford, UK) was used to assess abdominal intra-abdominal (visceral/medial) and subcutaneous fat depth as parameters of abdominal fat deposition [17]. The infants were lying in the supine position on a flat surface and the ultrasound probe was placed at a point where the midline of the transverse plane used for WC measurement intercepts with the xiphoid line. To measure visceral depth, the probe was placed on the longitudinal plane with a probe depth of 6 or 7 cm, and was defined as the distance between the peritoneal boundary and the lumbar vertebrae. Subcutaneous abdominal fat depth was measured with the probe on the transverse plane with a probe depth of 4 or 5 cm, and defined as the distance between the bottom of the cutaneous layer and the linea alba, the fibrous sheath lining the anterior abdominal wall.

**Figure 2:**
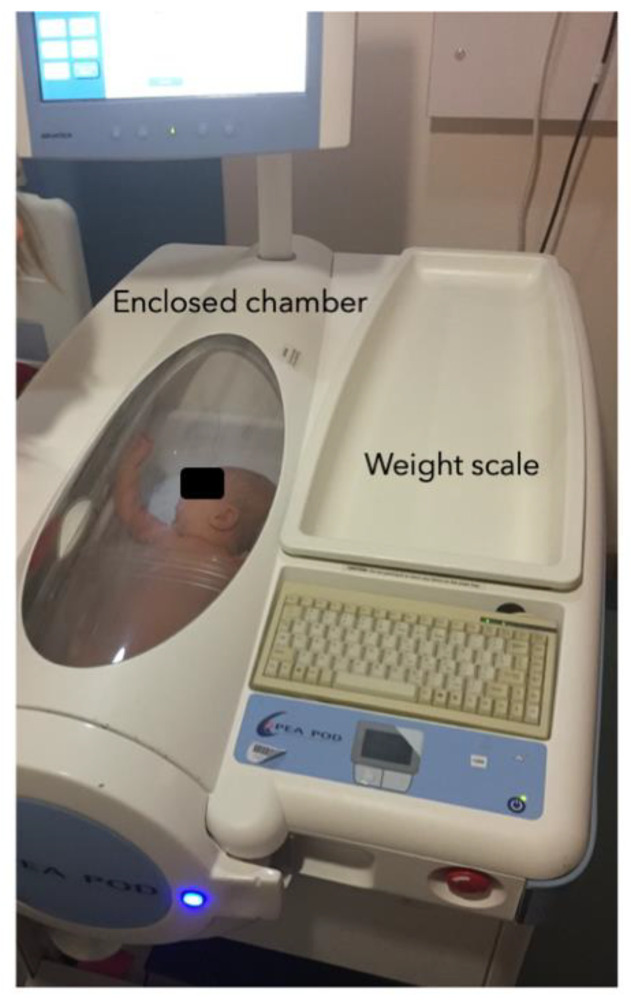
Infant body composition measurement using ADP-Pea Pod in CBGS-BF.

To assess infant total body fat- and fat-free mass, ADP using the Pea Pod system (Life measurement Inc., Concord, CA, USA) was performed at 6 weeks and 3 months. ADP Pea Pod is a safe and non-invasive procedure and infants were lain supine and naked inside the enclosed chamber (Figure 2). ADP Pea Pod assumes a two-component model of body composition (fat- and fat-free components) and is considered to be a criterion method for measuring infant body composition [18].

### 2.2. Biological Sample Collection

Dried blood spots (DBS) and a small amount of blood for extraction of plasma were sampled from heel prick at all research visits. Stool samples were collected at each visit from 2 weeks until 12 months (in total 5 visits) for microbiota analysis. To allow consideration of modifiers of microbiota composition, detailed data were gathered via structured questionnaires on exposures to pro/prebiotics, antibiotics, antifungals, and steroids in the 14 days before each stool sample.

Liquid hindmilk was collected at each visit from birth until 12 months of age if the mothers were still breastfeeding. Milk was hand- or pump-expressed after feeding the baby during the actual research visit, or in 7 days prior or after visit. To collect liquid BM for microbiota analysis at 6 weeks of the infant’s age, complete milk expression was taken place from one breast, using a breast pump and sterile milk collection unit provided by the research team. To ensure the sterile procedure, the breast was first cleaned using antiseptic soap and dried with sterile paper towels.

Analysis of non-sterile collected BM composition included macronutrients (carbohydrate, fat, protein), butyrate, and human milk oligosaccharides (HMOs). Dried milk spots (DMS) were also collected for lipidomics analyses in each visit until mothers stopped breastfeeding.

### 2.3. BM Intake Volume Measurement

To measure the amount of BM consumed by infants between 4–6 weeks of age, mother–infant deuterium-oxide (^2^H_2_O) turnover technique was employed [19,20]. Baseline urine samples from both mother and infant were collected on day 0, after which the mother received an oral dose of 50 g of this ‘heavy’ water. Further daily urine samples from mother and baby were collected over a 14-day period.

**Figure 3:**
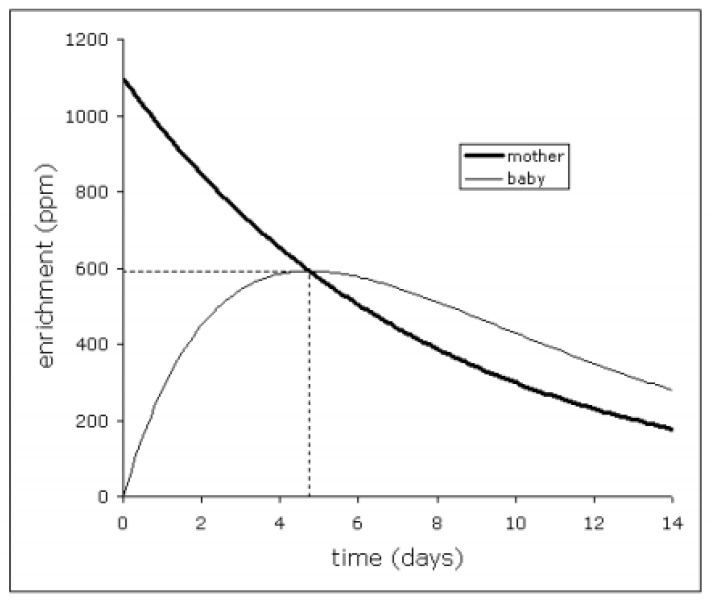
Isotope enrichment of mother’s and infant’s total body water for an exclusively BF infant (representative example pattern).

The volume of BM intake was then determined by measuring transfer of isotope enrichment from mother to her baby. After being administered, the deuterium-enriched tracer water was incorporated into the mother’s total body water (TBW) pool and passed onto her baby as BM. The amount of BM consumed by the baby could be calculated by analysing the rate of deuterium (^2^H) appearance in the baby’s urine and disappearance from the mothers urine (Figure 3). ^2^H enrichment in the urine samples was measured by isotope ratio mass spectrometry. The formulas and assumptions used for calculating BM intake were following those of Haisma et al. [20]. The experiment was conducted in a collaboration with the MRC Elsie Widdowson Laboratory and the results were displayed in L/day.

### 2.4. BM Macronutrient and Butyrate Analyses

Macronutrients and butyrate were analysed following the same protocol as used in the original CBGS [4,5]. Defrosted and homogenised liquid BM samples were mixed with CDC1_3_ solvent with 1:1 ratio. The resulting polar fraction was then used to measure butyrate concentration while the non-polar fraction was used to measure lipid concentrations using ^1^H-Nuclear magnetic resonance (NMR) spectra. Triglyceride (TG) served as a surrogate for total fat content since it contributes 95–98% of total BM lipid content. Meanwhile, the polar fraction of the BM sample was used to measure lactose, the most abundant BM carbohydrate, using ^1^H 1D nuclear Overhauser effect spectroscopy (NOESY). For protein, total nitrogen level was measured by the DUMAS method, and the protein factor conversion of 6.25 was used to calculate crude protein content. Atwater conversion factors were used to calculate the total metabolisable calorie content (TCC) of BM, taking energy contents of 4, 9, and 4 kcal/g for lactose, fat, and protein, respectively, and was expressed in kcal/100 mL. BM nutrient density was calculated as macronutrient content as % of TCC, i.e., %carbohydrate, %fat, %protein.

### 2.5. Human Milk Oligosaccharides Measurement

Human milk oligosaccharides (HMOs) were quantified from liquid BM samples obtained from 2 weeks to 12 months of age. Briefly, each milk sample was diluted and filtered, and its oligosaccharides were quantified by high-pH anion-exchange chromatography using a Thermo Scientific Dionex ICS-5000+ system with a pulsed amperometric detector (HPAEC-PAD) [21]. A selection of the most abundant and represented HMOs was chosen, consisting of 2′-fucosyllactose (2′-FL), 3-fucosyllactose (3-FL), lacto-N-fucopentaose I (LNFP I), lacto-N-tetraose (LNT), and lacto-N-neotetraose (LNnT) as neutral HMOs, and 3′-sialyllactose (3′-SL) and 6′-sialyllactose (6′-SL) as acidic HMOs.

In brief, each sample was prepared using the “dilute-and-shoot” method and analysed using HPAEC-PAD in duplicates. During sample dilution, five concentrations of the standards of each of the 7 HMO species being studied were spiked into a 6-week milk sample. Recovery was determined by calculating the ratio between the measured and theoretical spiked quantities. To assess repeatability, the same samples were injected 5 times and the coefficient of variation was calculated. For each HMO species, the limit of detection and the limit of quantification were empirically decided if the resulting concentration could produce a signal-to-noise ratio of 3:1 and 6:1, respectively [21].

### 2.6. Fucosyltransferase 2 (FUT2) Genotyping Study

*Fucosyltransferase 2 (FUT2)* genotyping study was performed on maternal and infant saliva. The single nucleotide polymorphism (SNP) target used in this study was rs516246 and the secretor phenotype was defined by homozygous G/G or heterozygous A/G genotypes, whereas homozygous A/A indicated the non-secretor phenotype [22].

### 2.7. Microbiota Analysis

Bacterial composition in maternal BM and infant stool was analysed by sequencing of the 16S ribosomal RNA (rRNA) genes. All procedures, including DNA extraction, PCR amplification, library preparation, and sequencing of the V3–V4 region of the 16S rRNA genes on a MiSeq sequencer (Illumina Inc., San Diego, CA, USA), were performed using a standard protocol with established quality control [23].

## 3. Results

This study recruited 119 infants at birth in total, of whom 94 were eligible for follow-up as receiving solely BM in the first 6 weeks of life. Of these, 14 infants were introduced to mixed-feeding between 6 and 12 weeks, 52 between 3 and 6 months, and the remaining 28 continued exclusive breastfeeding for at least 6 months. Up to date, all infants have completed follow-up to 12 months. The 24- and 36 months visits are ongoing.

The overall aim of the study is to assess the associations between human milk components and their intakes, with infant growth, weight gain, and changes in body composition. The primary outcomes are changes in age- and sex-standardised scores for infant length, weight, fat mass and fat-free mass between birth to age 12 months. We highlight below some of the key questions to be addressed.

### 3.1. The Relationship between Exclusive Breastfeeding and Infant Growth

We hypothesize that there is a bidirectional relationship between exclusive breastfeeding and infant growth. In this study, growth assessments and BM intake volumes were measured at age 6 weeks, while all infants were still exclusively breastfed. We conjecture that slower weight between birth and 6 weeks and lower BM intake volumes at 6 weeks will predict subsequent earlier introduction of infant formula and/or complementary foods. Conversely, we expect that earlier introduction of infant formula will predict subsequent faster weight gain. Few other infant cohort studies have collected sufficiently repeated assessments of growth and feeding to address such questions.

### 3.2. BM Intake Volume, BM Macronutrient Composition, and Infant Growth

In line with the above findings, we speculate negative associations between BM intake volumes at age 6 weeks and subsequent infant growth and adiposity gains, that could persist until 36 months.

We will also explore specific BM nutrient intakes as potential regulators of infant weight gain. To do this, associations between each BM macronutrient and BM intake volume will be investigated to highlight the importance of considering BM nutrient intakes when examining infant weight gain nutritional drivers, as opposed to simply BM nutrient contents.

Our previous publication and other studies have reported positive associations bet-ween BM lactose (representing carbohydrate) and protein with infant weight gain and adiposity [4,13,24,25,26]. BM fat intakes were also inversely associated with those growth para-meters in the original CBGS [4]. We are inclined to reproduce the same examinations in the CBGS-BF, supplemented by BM nutrient intakes analyses. We predict similar results for lactose and protein in this study, but not with fat as we collected hind-milk samples that might reduce interindividual BM fat content variations.

### 3.3. Factors Influencing HMOs Abundance and Its Relation to Infant Growth

By acting as act as soluble prebiotics and contributing to the establishment of desirable infant gut microbiota, HMOs have been evidence to promote overall infant health and growth via protection from infections and obesity [27,28]. However, the evidence of HMOs determinant factors as well as their effects during infancy is still scarce, inconclusive, and confounded by many factors, especially the amount of intake by infants.

Maternal *FUT2* polymorphism is reported to be the major determining factor in HMOs diversity and abundance. *FUT2* secretor and non-secretor mothers have distinct HMO profiles, with predominant 2′fucosyllactose (2′FL) and lacto-N-fucopentaose I (LNFPI) among secretors [27,29].

In this study, we attempt to examine the pre-, ante-, and postnatal influencing factors of HMOs and the association of HMOs intake and infant growth in the first year of life.

### 3.4. Infant Gut and BM Microbiota

We are planning to observe the diversity and development of infant gut microbiota over time, especially in relation to BM microbiota, breastfeeding duration, and HMOs. We hypothesize that exclusive breastfeeding duration and HMOs intakes influence bacterial abundance and diversity in infant gut microbiota, providing important insights into the relationship between mother’s milk and infant gut microbiome.

## 4. Discussion

Although data collection and sample analyses in the CBGS-BF are still ongoing, several findings have already emerged.

First, HMO abundances were prominently affected by stages of lactation and maternal genotype. Two HMO species, 3-FL and 3′-SL, increased in concentration as the lactation progressed while the others, including 2′-FL, LNFP I, LNT, LNnT, and 6′-SL decreased with time. Maternal *FUT2* genotype predicted the profile of oligosaccharides secreted in BM, with marked differences in BM concentrations of 2′-FL, 3-FL, LNFP I and LNT between secretors and non-secretors [21].

Second, the use of ADP-PEA Pod in the study has enabled the derivation of new prediction equations to estimate body composition during infancy. Infant sex, postnatal age at measurement, weight, length, and skinfold thicknesses were modelled to develop infant fat- and fat-free mass prediction equations against ADP-PEA Pod as the criterion. Recently, these have been validated in an independent study. The new prediction equations resulted in better validity and smaller bias compared to previously available equations that were based on measurements made only at birth [30].

The main strength of the study is the design of CBGS-BF that involves comprehensive and longitudinal data collection, including prenatal questionnaires and food diaries, diverse biological sample collection, and thorough anthropometry and body composition measurements. Measurements of BM intake volume using deuterium-labelled water, detailed assessment of BM composition including oligosaccharides and other components, and BM and infant gut microbiota profiles allow a unique depth of investigation into the mechanisms that link breastfeeding to healthy patterns of infant growth and weight gain.

Limitations of the study include the use of a single site with predominant White Caucasian population, which could limit the applicability of the results to more diverse populations. The application of stringent recruitment criteria as well as exclusion of infant-mother pairs who did not exclusively breastfeed for at least 6 weeks has advantages and disadvantages. While this focussed study sample minimises the confounding effects of many environmental factors, it limited the number of subjects eligible for inclusion.

## 5. Conclusions

The CBGS-BF aimed primarily to carry out extensive profiling of breastmilk intakes and composition in relation to infancy growth. Anonymised data can be made available to other researchers through collaborative agreements. The CBGS-BF investigators welcome formal or informal proposals and will consider these at their quarterly meetings. Prof Ken Ong (Ken.Ong@mrc-epid.cam.ac.uk).

## Figures and Tables

**Table 1:** Data, anthropometry, and biological samples collection during each visit in the Cambridge Baby Growth and Breastfeeding Study (CBGS-BF).

	Birth	2w	6w	3M	6M	12M	24M	36M
Consent and recruitment	+							
Collection of perinatal questionnaire and parental demographics	+							
Infant’s anthropometry and body composition								
Weight, length, head circumference, waist circumference	+	+	+	+	+	+	+	+
Skinfold thicknesses	+	+	+	+	+	+	+	
Abdominal ultrasound			+	+	+	+	+	
ADP-Pea Pod			+	+				
Infancy questionnaires								
Allergy, infection/antibiotics exposure, probiotic exposure, feeding history	+	+	+	+	+	+		
Food diary					+	+	+	+
Biological samples								
Infant’s stool sample for gut microbiota		+	+	+	+	+		
Sterile collection of BM for milk microbiota			+					
Other (non-sterile collection of) BM liquid sample and DMS	+	+	+	+	+	+		
Mother’s and infant’s urine for BM intake volume measurement			+					
			(4–6 w)					
Infant’s blood sample (DBS and small amount of plasma)	+	+	+	+	+	+		
Parents’ and infant’s saliva (DNA trio)					+			

w = weeks, M = months, BM = breast milk, ADP = air displacement plethysmography, DMS = dried milk spot, DBS = dried blood spot.

## Data Availability

Anonymised data can be made available to other researchers through collaborative agreements. Please contact Prof Ken Ong (Ken.Ong@mrc-epid.cam.ac.uk).
